# Phenotypic and Genetic Characterization of Temperature-Induced Mutagenesis and Mortality in *Cupriavidus metallidurans*

**DOI:** 10.3389/fmicb.2021.698330

**Published:** 2021-07-09

**Authors:** Rob Van Houdt, Joachim Vandecraen, Wietse Heylen, Natalie Leys, Pieter Monsieurs, Ann Provoost, Abram Aertsen

**Affiliations:** ^1^Microbiology Unit, Belgian Nuclear Research Centre (SCK CEN), Mol, Belgium; ^2^Department of Microbial and Molecular Systems, Faculty of Bioscience Engineering, KU Leuven, Leuven, Belgium

**Keywords:** *Cupriavidus*, growth temperature, amino acids, nitrogen, osmotic pressure

## Abstract

*Cupriavidus metallidurans* strains display a decreased viability when incubated in rich medium at a temperature of 37°C compared to their normal growth temperature of 30°C, a phenomenon coined “temperature-induced mortality and mutagenesis” (TIMM). To scrutinize this aberrant phenotype further, the contributions of specific inducers and protective agents were determined. Different growth media, including lysogeny broth (LB) and Schatz, and components, including casamino acids, in particular amino acids (proline, cysteine, glycine, glutamine, leucine, histidine and phenylalanine) and ammonium, were found to induce TIMM at 37°C. Sorbitol was found to counteract TIMM. Furthermore, although TIMM is well conserved within the *C. metallidurans* species, multiple and strain-specific TIMM inducers exist. Twenty-nine percent of the TIMM survivors inherited resistance to TIMM. Whole-genome sequencing of two resistant derivatives revealed an important role of an uncharacterized oxidoreductase, indicating putative metabolic poisoning when grown in high-concentration nitrogen-containing media at 37°C.

## Introduction

Type strain *Cupriavidus metallidurans* CH34, isolated from a non-ferrous metallurgical plant in Belgium (Mergeay et al., 1978), is mostly studied because of its resistance to numerous metals (Mergeay and Van Houdt, 2015). In addition, Mergeay et al. (1985) described an intriguing phenomenon of decreased viability (with typical survival frequencies ranging between 10^–3^ and 10^–5^) when *C. metallidurans* CH34 is grown in rich medium [i.e., lysogeny broth (LB)] at an increased temperature of 37°C compared to the normal growth temperature of 30°C. It was hypothesized that the phenomenon corresponded to a temperature-induced mutator phenotype as up to 80% of the surviving colonies exhibited one or more aberrant phenotypes, including auxotrophy for lysine, threonine, proline or serine; loss of autotrophy (growth on H_2_ and CO_2_); loss of nitrate utilization; loss of ammonium utilization as a nitrogen source; loss of tyrosine as a carbon source; and loss of cobalt/zinc resistance (Sadouk and Mergeay, 1993). Therefore, they coined this phenomenon as “temperature-induced mortality and mutagenesis” (TIMM). Next to rich LB medium, a similar phenotype was observed when methionine was specifically added to a mineral medium (Van der Lelie et al., 1992). Interestingly, most TIMM survivors, although isolated at a higher temperature, remained susceptible to an increase in temperature (Mergeay, 2000).

Growing *C. metallidurans* CH34 in LB medium at 37°C for 1 h had no effect on a variety of measured physiological parameters, including cell size, membrane permeability, membrane potential, intracellular esterase activities, intracellular pH and production of reactive oxygen species (ROS) (Baatout et al., 2005). However, during prolonged growth at 37°C, morphological changes such as filamentous cell formation, bead-like chains and cell clusters were induced (Arroua et al., 2014). This aberrant growth was hypothesized to be caused by impaired peptidoglycan synthesis or processing. Moreover, when cultures grown in LB medium were shifted from permissive to non-permissive temperature, a full growth arrest was induced after approximately two generations, which was probably caused by the depletion of an essential cell component and/or activity (Arroua et al., 2014). In addition, Arroua et al. (2014) suggested that the TIMM phenotype may be part of a more general stress response as the same morphological changes could be induced by sodium hypochlorite.

In this study, 22 different *C. metallidurans* strains were tested for their susceptibility to TIMM, and the TIMM phenotype was further scrutinized by testing different growth media for their ability to induce mortality at the restrictive temperature of 37°C. The potential mitigation of TIMM induction by adding a non-ionic osmolyte or ROS scavengers was scrutinized as well as the possible stable inheritance of TIMM resistance by *C. metallidurans* CH34.

## Materials and Methods

### Strains, Media and Culture Conditions

Bacterial strains and plasmids used in this study are listed in Tables 1, 2. *C. metallidurans* strains were routinely cultured at 30°C in Tris-buffered mineral medium (6.06 g/L Tris/HCl, 4.68 g/L NaCl, 1.49 g/L KCl, 1.07 g/L NH_4_Cl, 0.43 g/L Na_2_SO_4_, 0.2 g/L MgCl_2_⋅6H_2_0, 0.03 g/L CaCl_2_⋅2H_2_0, 0.04 g/L Na_2_HPO_4_⋅2H_2_O, 4.8 mg/L Fe(III)(NH_4_)citrate, 144 μg/L ZnSO_4_⋅7H_2_O, 99 μg/L MnCl_2_⋅4H_2_O, 62 μg/L H_3_BO_3_, 190 μg/L CoCl_2_⋅6H_2_O, 17 μg/L CuCl_2_⋅2H_2_O, 24 μg/L NiCl_2_⋅6H_2_O, and 36 μg/L Na_2_MoO_4_⋅2H_2_O) supplemented with 0.2% (w/v) sodium gluconate (MM284) as described previously (Mergeay et al., 1985). *Escherichia coli* strains were routinely cultured at 37°C in LB. Liquid cultures were grown in the dark on a rotary shaker at 150 rpm; for culturing on agar plates, 2% agar (Thermo Scientific, Oxoid) was added. *C. metallidurans* strains were also cultured on Schatz mineral salt medium, as described previously (Schatz and Bovell, 1952), supplemented with 0.2% (w/v) sodium gluconate. When appropriate, the following chemicals (Sigma-Aldrich or Thermo Scientific) were added to the growth medium at the indicated final concentrations: casamino acids (CA) (0.5% (w/v)), individual L-amino acids (0.5% (w/v)), sorbitol (0.44 M), NaCl (0.5–1% (w/v)), mannitol (50 mM), pyruvic acid (10 mM), kanamycin (50 and 1,500 μg/ml for *E. coli* and *C. metallidurans*, respectively), chloramphenicol (30 μg/ml), 5-bromo-4-chloro-3-indolyl β-galactopyranoside (X-Gal; 40 μg/ml), and isopropyl β-D-1-thiogalactopyranoside (IPTG; 0.1 mM).

**TABLE 1:** Strains and plasmids used in this study.

**Strain or plasmid**	**Genotype/relevant characteristics^1^**	**References**
**STRAIN**		
****Cupriavidus metallidurans****		
CH34	pMOL28, pMOL30, TIMM^*S*^	Mergeay et al., 1985
CH34-TIMM^*R1*^	CA TIMM^*R*^-derivative of CH34	This study
CH34-TIMM^*R2*^	CA TIMM^*R*^-derivative of CH34	This study
CH34-Km^*R*^	CH34 harboring a EZ-Tn5 <*KAN*−2> in Rmet_0221 coding for a conserved hypothetical protein, Km^*R*^	This study
CH34 Δ1009	CH34 Rmet_1009::*tet*, Tc^*R*^	This study
CH34-TIMM^*R1*^ Δ1009	CH34-TIMM^*R1*^ Rmet_1009::*tet*, Tc^*R*^	This study
CH34-TIMM^*R2*^ Δ1009	CH34-TIMM^*R2*^ Rmet_1009::*tet*, Tc^*R*^	This study
***Escherichia coli***		
DG1	Strain for cloning and plasmid preparation	Eurogentec, B
MFD*pir*	MG1655 RP4-2-Tc::[Δ*Mu1::aac(3)IV*-Δ*aphA*-Δ*nic35*-Δ*Mu2::zeo*] Δ*dapA*::(*erm-pir*) Δ*recA*	Ferrieres et al., 2010
**PLASMID**		
pACYC184	p15A ori, Cm^*R*^, Tc^*R*^	Lab collection
pK18mob	pMB1 ori, Mob+, *lacZ*, Km^*R*^	Schafer et al., 1994
pK18mob-1009::*tet*	*tet* gene flanked by 1 kb DNA region upstream and downstream of Rmet_1009, Km^*R*^, Tc^*R*^	This study

*^1^TIMM^*S*^: TIMM susceptible; TIMM^*R*^: TIMM resistant; CA: casamino acids; Km^*R*^: kanamycin resistant; Cm^*R*^: chloramphenicol resistant; Tc^*R*^: tetracycline resistant.*

**TABLE 2:** Survival frequency and isolation site/place of the different *C. metallidurans* strains used in this study as determined by the ratio of viable count on LB agar at 37 to 30°C.

**Strain**	**Survival freq.**	**Isolation site (country)**	**References**
CH34	3.82 ± 0.52 × 10^–5^	Decantation tank, zinc factory (BE)	Mergeay et al., 1985
AE104	1.87 ± 0.31 × 10^–5^	CH34 cured from pMOL28 and pMOL30	Mergeay et al., 1985
AE126	1.68 ± 0.34 × 10^–4^	CH34 cured from pMOL30	Mergeay et al., 1985
AE128	1.69 ± 0.26 × 10^–4^	CH34 cured from pMOL28	Mergeay et al., 1985
KT01	3.38 ± 0.46 × 10^–5^	Wastewater treatment plant (DE)	Timotius and Schlegel, 1987
KT02	2.14 ± 0.22 × 10^–5^	Wastewater treatment plant (DE)	Schmidt et al., 1991
KT21	2.15 ± 0.18 × 10^–5^	Wastewater treatment plant (DE)	Timotius and Schlegel, 1987
CH42	4.38 ± 0.46 × 10^–5^	Polluted sediments, zinc factory (BE)	Brim et al., 1999
CH79	3.18 ± 0.37 × 10^–6^	Polluted sediments, zinc factory (BE)	Brim et al., 1999
AS39	6.85 ± 0.56 × 10^–5^	Mine tailings (CG)	Diels and Mergeay, 1990
AS167	2.63 ± 0.24 × 10^–4^	Mine tailings (CG)	Brim et al., 1999
AS168	1.05 ± 0.12 × 10^–4^	Mine tailings (CG)	Diels and Mergeay, 1990
31A	5.07 ± 0.82 × 10^–5^	Galvanization tank, metal factory (DE)	Schmidt et al., 1991
SV661	1.13 ± 0.21 × 10^–4^	Non-ferrous industry (BE)	Diels and Mergeay, 1990
ccug38404	1.85 ± 0.32 × 10^–4^	Human urine	CCUG^1^
ccug43015	1.96 ± 0.46 × 10^–4^	Human cerebrospinal fluid	CCUG
ccug45957	1.73 ± 0.24 × 10^–4^	Pharmaceutical industry (SE)	CCUG
H1130	4.76 ± 0.36 × 10^–5^	Clinical Isolate	Langevin et al., 2011
NE12	4.03 ± 0.28 × 10^–5^	Cleanroom Kennedy Space Center (United States)	Mijnendonckx et al., 2013
NA1	9.5 0 ± 0.54 × 10^–3^	Water storage system (ISS)	Mijnendonckx et al., 2013
NA2	8.70 ± 0.41 × 10^–3^	Contingency water container (ISS)	Mijnendonckx et al., 2013
NA4	2.61 ± 0.23 × 10^–4^	Water recovery system (ISS)	Mijnendonckx et al., 2013

*^1^CCUG: Culture Collection, University of Gothenburg, Sweden.*

### TIMM Assay and Isolation of TIMM-Resistant Mutants

In this study, unless cited otherwise, LB agar plates were used to study the TIMM phenotype. The TIMM assay comprised cultivating a 10-fold serial dilution of stationary-phase cells on agar plates at 30°C (permissive temperature) and 37°C (restrictive temperature). After an incubation time of 2 days, the survival frequency was calculated as viable cell count at 37°C divided by viable cell count at 30°C. A survival frequency of 10^–2^ or less was scored as a positive TIMM phenotype. Additional TIMM assays were performed to determine the frequency of inherited TIMM resistance. Ten independent *C. metallidurans* CH34 lines were cultivated in MM284 at 30°C up to the stationary phase and 10^9^ cells were pelleted. One-hundred-microliter aliquots of a 10-fold serial dilution in saline were spread on MM284 agar plates containing 0.5% (w/v) L-phenylalanine and incubated for 2 days at 37°C. Next, 10 survivors of each independent line, thus totaling 100 survivors for each strain, were purified at 30°C. Finally, these survivors were retested for TIMM resistance by being spread on different agar plates, including LB, Schatz medium and MM284 containing 0.5% (w/v) L-phenylalanine or CA.

### Fitness Analysis

The fitness of stable TIMM-resistant CH34 mutant strains was compared with that of the parental CH34 by culturing equally mixed cell suspensions in triplicate. To enable selection, a reference *C. metallidurans* CH34 strain, i.e., CH34-Km^*R*^, containing a kanamycin resistance cassette in gene Rmet_0221, coding for a hypothetical protein, was used in the competition assays. The different strains, i.e., CH34-TIMM^*R1*^, CH34-TIMM^*R2*^, CH34 and CH34-Km^*R*^ (Table 1), were first individually cultivated up to the stationary phase (MM284, 30°C) and subsequently used to inoculate tubes with 4 ml MM284 (biological triplicate) with mixed cultures of CH34-Km^*R*^ and the TIMM-resistant mutant or parent, respectively, at a final concentration of 10^5^ cells/ml. The tubes were incubated with shaking at 30°C, and 20 μl aliquots were withdrawn at different time points. Cell enumeration by total viable count was performed by spreading 100 μl of a serial 10-fold dilution in sterile PBS on MM284 agar and counting colonies after 2 days at 30°C. Plates with 30–300 colonies were selected as master plates for replica plating onto MM284 + 1,500 μg/ml kanamycin agar plates, a condition where only CH34-Km^*R*^ will survive, and the CFU/ml was determined again after 2 days of incubation at 30°C. The fitness of the different strains compared to the reference strain was determined as the ratio of CFU/ml on MM284 to MM284 + 1,500 μg/ml kanamycin.

### Construction Insertional Inactivation Mutant

The Rmet_1009 gene including the 1-kb region upstream and downstream was amplified by PCR (Phusion High-Fidelity DNA polymerase, Thermo Scientific) with the primer pair Rmet_1009_5′-FW/_3′-RV providing *Mph*1103I/*Eco*RI recognition sites. This PCR product was cloned as a *Mph*1103I/*Eco*RI fragment into *Pst*I/*Eco*RI-digested pK18mob. The resulting pK18mob-1009 plasmid from an *E. coli* DG1 transformant selected on LB Km50 was further confirmed by sequencing prior to amplifying of the flanking sequences of Rmet_1009 by inverse PCR (Phusion High-Fidelity DNA polymerase) with the primer pair Rmet_1009_5′-RV/_3′-RW, providing *Spe*I/*Bsp*TI restriction sites. At the same time, the *tet* gene from pACYC184 was amplified by PCR (Phusion High-Fidelity DNA polymerase) with the primer pair Tet_Fw-Rv, providing *Spe*I/*Bsp*TI restriction sites. Afterward, this PCR product was cloned as a *Spe*I/*Bsp*TI fragment into the former inverse PCR product. The resulting pK18mob-1009::*tet* plasmid from an *E. coli* DG1 transformant selected on LB Tc^20^ Km^50^ was further confirmed by sequencing prior to conjugation (biparental with *E. coli* MFD*pir* as a donor) to *C. metallidurans* CH34, CH34-TIMM^*R1*^ and CH34-TIMM^*R2*^. The resulting transformants selected on MM284 Tc^20^ were replica-plated on MM284 Tc^20^ and MM284 Km^1500^. Transformants resistant to Tc^20^ but sensitive to Km^1500^ were further confirmed by sequencing and were designated CH34 Δ1009, CH34-TIMM^*R1*^ Δ1009, and CH34-TIMM^*R2*^ Δ1009. All primers used in this study are listed in Supplementary Table 1.

### Genome Sequencing and Analysis

Whole-genome sequencing of two CA TIMM-resistant CH34 derivatives, i.e. strains CH34-TIMM^*R1*^ and CH34-TIMM^*R2*^ (Table 1), was performed to identify mutations responsible for the stable inheritance of TIMM resistance. The strains were cultivated by inoculating 4 ml LB at 30°C, and total DNA was extracted using the QIAamp DNA Mini Kit (Qiagen, Netherlands). The quantity and quality of extracted DNA were measured using a NanoDrop^TM^ 1000 spectrophotometer (Thermo Scientific, United States). Ten micrograms of DNA were sent for Illumina sequencing (BaseClear, Netherlands). Point mutations (SNPs and small indels) and large insertions and deletions (>200 bp) were identified as described by Mijnendonckx et al. (2019). Sequencing data are available within the Sequencing Read Archive (SRA) of NCBI under the bioProject PRJNA641768.

## Results

### TIMM Is Well Conserved Among *C. metallidurans* Strains

As detailed in the introduction, *C. metallidurans* CH34 exhibits a decreased viability when cultivated on nutrient-rich agar plates at 37°C, and up to 80% of the surviving colonies exhibited one or more aberrant phenotypes, including loss of autotrophy; auxotrophy for lysine, threonine, proline, or serine; or loss of ammonium utilization as a nitrogen source (Mergeay et al., 1985; Van der Lelie et al., 1992; Sadouk and Mergeay, 1993; Mijnendonckx et al., 2013). Besides strain CH34, other strains isolated from metal-contaminated industrial environments showed this TIMM phenotype as well (Brim et al., 1999). Here, we confirmed this phenotype for a subset of these strains (KT01, KT02, KT21, CH42, CH79, AS39, AS167, AS168, 31A and SV661), which were recently included in a comparative genomic hybridization study to type strain CH34 (Van Houdt et al., 2012), and demonstrated it for more recently isolated strains from anthropogenic environments not typified by metal contamination (ccug45957, NE12, NA1, NA2 and NA4) and from human infections (ccug38404, ccug43015 and H1130) (Table 2). All these *C. metallidurans* strains displayed increased mortality on LB agar at 37°C with a frequency of survivors ranging from 10^–4^ to 10^–6^ (Table 2). In addition, the CH34 derivatives cured from one or both megaplasmids (AE104, AE126 and AE128) displayed increased mortality (Table 2).

### TIMM-Mediated Cell Mortality After Prolonged Exposure at 37°C

**FIGURE 1:**
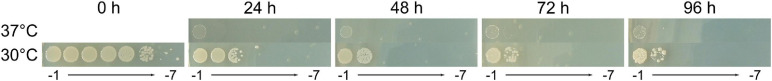
Tenfold serial dilution of a stationary phase culture of CH34 spotted on LB agar plates and incubated at 37°C (upper row) for 0, 24, 48, 72 and 96 h before shifting to an 48 h incubation period at 30°C (lower row).

Survival of *C. metallidurans* CH34 under TIMM-inducing conditions was further assessed in the function of incubation time. At different time points post-plating, LB agar plates were shifted from restrictive to permissive temperatures, and the viability was determined after 2 days of incubation at 30°C following the shift from 37 to 30°C (Figure 1). It was demonstrated that cell mortality was initiated during prolonged exposure to 37°C. Approximately, a 4-log reduction in viability was observed after 24 h of exposure. Viability further decreased 1 log (48 h at 37°C) and remained more or less stable at 72 and 96 h. Thus, a reduced viable cell count during TIMM conditions resulted from cell death and not merely by a decreased or anomalous growth rate.

### TIMM-Inducing and Protective Media

Previously, TIMM induction was observed when cells were incubated at 37°C on LB agar plates or on MM284 agar plates supplemented with L-methionine (Van der Lelie et al., 1992). Here, we scrutinized different growth media and possible protective agents for their ability to either induce or inhibit TIMM at 37°C (Table 3). In contrast with previous observations, cells incubated on MM284 containing 0.5% (w/v) L-methionine did not display TIMM. Modifications of LB medium (a mixture of 1% (w/v) sodium chloride, 0.5% (w/v) yeast extract and 1% (w/v) peptone) were made in order to identify and evaluate specific TIMM inducers. Media composed of only sodium chloride with either peptone or yeast extract were tested, and both induced TIMM (Table 3). Both peptone and yeast extract have a high peptide and amino acid content with yeast extract also comprising different vitamins and sugars. Subsequently, the role of amino acids was further analyzed using MM284 containing 0.5% (w/v) CA. CA, the hydrolysis product of casein, comprise small peptides and amino acids with the exception of tryptophan. *C. metallidurans* CH34 grown on MM284 containing 0.5% (w/v) CA showed a survival frequency at 37°C comparable to LB, confirming that amino acids play an important role in TIMM induction. In addition, other *C. metallidurans* strains, including NA1, NA2, NA4 and ccug43015, were tested, and CA promoted TIMM for all tested strains (data not shown).

**TABLE 3:** Different media and strains used in this study to evaluate TIMM induction (+ for induction and − for no induction) as determined by the ratio of viable count at 37 to 30°C.

**Medium**	**CH34**	**CH34-TIMM^*R1/2*^**
LB (1% NaCl, 0.5% YE, 1% P)^1^	+	+
0.5% YE + 1% NaCl	+	+
1% P + 1% NaCl	+	−
MM284 (0.5% NaCl, 0.2% NH_4_Cl)	−	−
MM284 + 0.5% CA^2^	+	−
MM284 + 0.5% Phe	+	−
MM284 + 0.5% Gly	+	−
MM284 + 0.5% Cys	+	−
MM284 + 0.5% Leu	+	−
MM284 + 0.5% Pro	+	−
MM284 + 0.5% Gln	+	−
MM284 + 0.5% His	+	−
MM284 − NaCl	+	−
MM284 + 0.5% NH_4_Cl	−	−
MM284 + 1% NH_4_Cl	+	−
MM284 + 0.5% NH_4_NO_3_	−	−
MM284 + 1% NH_4_NO_3_	+	−
MM284 + 1% NaCl	−	−
Schatz (0.1% NH_4_NO_3_)	+	+
Schatz + 1% NaCl	−	−
Schatz + 1% NaCl + 0.5% CA	+	−
+0.44 M sorbitol^3^	−	−

*^1^YE, yeast extract; P, peptone. ^2^CA, casamino acids. ^3^Addition of sorbitol completely inhibited TIMM induction in all known TIMM-inducing conditions.*

The use of CA as a sole carbon or nitrogen source or as an additional carbon/nitrogen nutrient to the available sodium gluconate and ammonium in MM284 presented no differential effect on TIMM induction. Next, all 20 amino acids were individually added to MM284 at a final concentration of 0.5% (w/v), and seven different amino acids induced TIMM (Tables 3, 4). Interestingly, not all *C. metallidurans* strains displayed the same sensitivity toward particular amino acids (Table 4), indicating strain-specific inducers and the possible involvement of multiple pathways. The individual amino acids that induced the TIMM phenotype for at least one of the *C. metallidurans* strains included proline, cysteine, glycine, glutamine, leucine and histidine with only phenylalanine being an inducer for all tested strains (Table 4).

**TABLE 4:** Effect of supplementing a specific amino acid to MM284 at a final concentration of 0.5% (w/v) on TIMM induction for different *C. metallidurans* strains (+ for induction and − for no induction) as determined by the ratio of viable count at 37 to 30°C.

**MM284 +**	**CH34**	**NA1**	**NA2**	**NA4**	**ccug43015**
**Non-polar**					
–	−	−	−	−	−
Ala	−	−	−	−	−
Gly	−	+	+	+	−
Ile	−	−	−	−	−
Leu	−	−	−	+	−
Met	−	−	−	−	−
Pro	+	+	−	+	−
Val	−	−	−	−	−
**Polar uncharged**					
Asn	−	−	−	−	−
Cys	+	+	+	−	+
Gln	+	+	+	−	−
Ser	−	−	−	−	−
Thre	−	−	−	−	−
**Polar charged**					
Arg	−	−	−	−	−
Asp	−	−	−	−	−
Glu	−	−	−	−	−
His	+	−	−	+	−
Lys	−	−	−	−	−
**Aromatic**					
Phe	+	+	+	+	+
Trp	−	−	−	−	−
Tyr^1^	ND*	ND*	ND*	ND*	ND*

*^1^ND: not determined as addition of Tyr resulted in crystallization of the medium.*

Next, the effect of surplus inorganic nitrogen, i.e., NH_4_Cl and NH_4_NO_3_, on TIMM induction was tested. It was observed that a gradual increase in inorganic nitrogen induced TIMM, indicating that excess nitrogen resulted in aberrant survival at 37°C (Table 3). In addition, growth on a mineral medium with low salt concentrations, i.e. Schatz medium (Table 3), and on carbon dioxide and hydrogen gas (data not shown) also induced TIMM. None of these additions affected survival at 30°C.

Since the possible aberrant catabolism of nutrients at 37°C might lead to the production of ROS, the addition of ROS scavengers was evaluated in the search of TIMM protective agents. Although addition of mannitol or pyruvic acid to MM284 containing 0.5% (w/v) L-phenylalanine exerted a positive effect at 37°C, a complete mitigation of the TIMM phenotype was not reached, indicating that ROS production probably does not, at least solely, cause the observed mortality (data not shown). In a next step, the effect of a non-ionic compatible osmolyte was assessed. Such compounds can exert favorable effects on macromolecule–solvent interactions, e.g. via stabilizing the cell membrane and increasing the protein thermal stability (Yancey, 2001). Addition of sorbitol, which cannot be used as a carbon source (Van Houdt et al., 2018), completely inhibited TIMM induction in all known TIMM-inducing conditions (Table 3). Finally, increasing the osmolarity of the mineral salt medium Schatz to levels approximating MM284 mitigated TIMM induction. The impact of the osmotic potential was further demonstrated by the fact that MM284 deprived of NaCl promoted TIMM. Is important to note that addition of CA to Schatz containing 1% NaCl again promoted TIMM, indicating that osmolarity and a nutrient-rich medium, although both counteracted by the addition of sorbitol, probably promoted TIMM via a different pathway.

### Characterization of Stable Inherited TIMM Resistance

**FIGURE 2:**
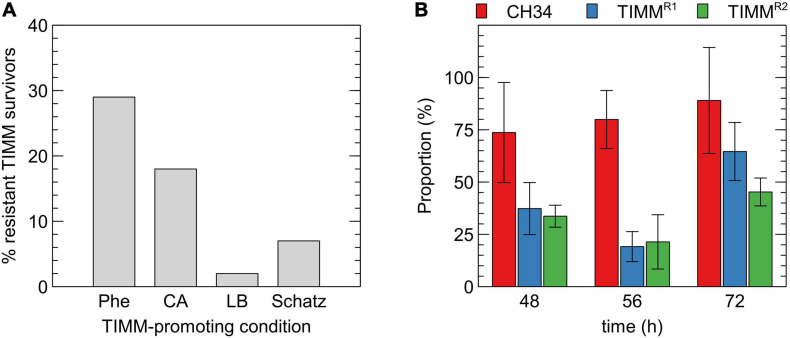
Characterization of TIMM survivors. **(A)** Inherited resistance of 100 TIMM survivors, originally isolated on MM284 containing 0.5% (w/v) L-phenylalanine, to different TIMM-promoting conditions as determined by the ratio of viable count at 37 to 30°C. Phe: L-phenylalanine, CA: casamino acids. **(B)** Relative fitness of the parental strain CH34 (red), CH34-TIMM^*R1*^ (blue) and CH34-TIMM^*R2*^ (green) in MM284 at 30°C, as determined by the ratio of viable count of the strains to CH34-Km^*R*^ in function of time.

*Cupriavidus metallidurans* CH34 was subjected to a directed evolution experiment to determine the frequency of inherited TIMM resistance in initial TIMM survivors randomly selected after 2 days of incubation at 37°C. Ten independent lines were plated on MM284 with 0.5% (w/v) L-phenylalanine and incubated at 37°C for 48 h. The latter medium was chosen because of its basic and defined composition and because L-phenylalanine induced TIMM in all *C. metallidurans* strains. Next, 10 TIMM survivors of each independent line, totaling 100 TIMM survivors, were purified at 30°C and subsequently retested for their TIMM resistance on L-phenylalanine, CA, LB and Schatz medium. The frequency of inherited resistance for *C. metallidurans* CH34 was 29% with lower cross-resistance to other TIMM-inducing media (Figure 2A).

### Analysis of TIMM-Resistant CH34 Derivatives

Two stable TIMM-resistant CH34 derivatives, i.e. strains CH34-TIMM^*R1*^ and CH34-TIMM^*R2*^, from two independent lines were further analyzed (Table 1). The fact that TIMM survivors displayed a lower cross-resistance to other TIMM-inducing media indicated the possible involvement of numerous or additional pathways in the different growth media. Therefore, strains CH34-TIMM^*R1*^ and CH34-TIMM^*R2*^ were selected since they displayed inherited resistance to the applied stress (e.g., L-phenylalanine) and amino acids (CA) but not to LB and Schatz medium.

#### Reduced Fitness of TIMM-Resistant Derivatives in Non-restrictive Conditions

It was previously reported that a large fraction of the TIMM survivors, ranging from 2 to 42%, showed additional mutations resulting in other aberrant phenotypes (Mergeay et al., 1985; Sadouk and Mergeay, 1993; Mergeay, 2000; Mijnendonckx et al., 2013). A fitness analysis was performed to find out if throughout the selective TIMM condition, additional detrimental mutations occurred that hitchhiked with the mutations relieving the selective pressure. The fitness of strains CH34-TIMM^*R1*^ and CH34-TIMM^*R2*^ was compared to that of the CH34 parent in a co-cultured growth competition assay under controlled laboratory conditions (Figure 2B). Although the experiment displayed a high variation, it demonstrated that the parental strain exerted a fitness advantage over the mutants when grown in MM284 at 30°C, indicating that the TIMM-resistant derivatives probably harbored accompanying detrimental mutations leading to a decreased fitness at the tested non-selective conditions.

#### Stable TIMM-Resistant CH34 Derivatives Harbor Numerous Mutations

**FIGURE 3:**
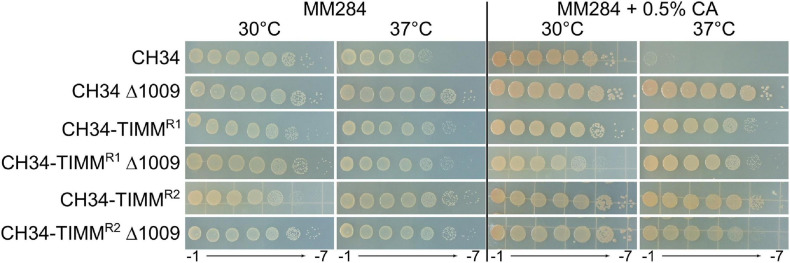
Tenfold serial dilution of stationary-phase cultures of CH34, CH34-TIMM^*R1*^ and CH34-TIMM^*R2*^, and their Rmet_1009 insertional knockout mutants spotted on MM284 agar plates with and without 0.5% (w/v) CA and incubated for 2 days at 30 and 37°C.

To further characterize the inherited TIMM resistance, whole-genome sequencing was performed. CH34-TIMM^*R1*^ and CH34-TIMM^*R2*^ harbored 14 and 11 mutations, respectively, including three common mutations (Table 5). Mutations were caused by insertions, deletions, and point and frameshift mutations (Table 5). Five mutations targeted genes encoded by either pMOL30 or pMOL28, but these are not likely to be responsible for the observed TIMM resistance as the plasmid-free derivative of CH34, i.e. strain AE104, could also evolve TIMM resistance. One large common deletion, approximately 60 kb, contained the *hox*/*cbb* locus and explained the observed loss of autotrophy of both strains. Loss of autotrophy is one of the most observed aberrant phenotypes identified under TIMM survivors but is not common to all TIMM survivors (Sadouk and Mergeay, 1993; Mergeay, 2000; Mijnendonckx et al., 2013). Only one other mutation was shared by both TIMM survivors, a single-nucleotide substitution in Rmet_1009, encoding an oxidoreductase subunit. An insertional knockout mutant was generated in CH34, CH34-TIMM^*R1*^ and CH34-TIMM^*R2*^. Inactivation of Rmet_1009 had no effect on TIMM resistance in CH34-TIMM^*R1*^ and CH34-TIMM^*R2*^ but resulted in acquisition of TIMM resistance in CH34 (Figure 3). The latter indicated an important role of Rmet_1009 in CA-induced TIMM.

**TABLE 5:** Identified mutations in CH34-TIMM^*R1*^ (R1) and CH34-TIMM^*R2*^ (R2).

**Replicon^1^**	**Gene^2^**	**Protein function**	**Mutation^3^**	**R1**	**R2**
CHR1	*hox*/*cbb*	Hydrogenotrophy	Δ 60 kb	+	+
CHR1	0862	Conserved hypothetical protein	INS IS*1088*	+	−
CHR1	1009	Oxidoreductase/nitroreductase	SNP	+	+
CHR1	1107	Putative membrane protein	INS IS*1088*	+	−
CHR1	1492	Fructose-bisphosphate aldolase	2 SNPs	+	−
CHR1	1563	Transposase IS*Rme12*	2 SNPs	+	−
CHR1	1885	Proteolytic subunit of ClpA-ClpP and ClpX-ClpP ATP-dependent serine proteases	SNP	−	+
CHR1	1886	Peptidyl-prolyl *cis*/*trans* isomerase	INS IS*1090*	−	+
CHR1	2409	Phosphatidylglyc erophosphate synthetase	INS IS*1088*	+	−
CHR1	6452	Hypothetical protein	2 SNPs	−	+
CHR2	3658	Type II secretion system protein	SNP	−	+
CHR2	4106	Acetone carboxylase α-subunit	SNP	+	−
CHR2	4757	Hypothetical protein	INS Tn*6048*	−	+
CHR2	4970	Formyl-CoA transferase	SNP	−	+
CHR2	4972	Tn*6048*	Δ 6 kb	−	+
CHR2	5469	Hypothetical protein	INS IS*1086*	+	−
CHR2	5806	Ferric citrate outer membrane transporter	INS IS*1088*	+	−
pMOL28	6321	Conserved hypothetical protein	+1 frameshift	+	+
pMOL28	6326	Conserved hypothetical protein	INS IS*1087B*	+	−
pMOL30	6072	Putative transcriptional regulator	SNPs	+	−
pMOL30	6072	Putative transcriptional regulator	INS IS*Rme15*	+	−
pMOL30	6097	2-Nitropropane dioxygenase	SNP	−	+

*^1^CHR1: chromosome; CHR2: chromid, pMOL28 and pMOL30 are megaplasmids. ^2^Locus tag (Rmet_XXXX). ^3^Δ: deletion, INS: insertion, SNP: single-nucleotide polymorphism.*

## Discussion

Elevating the temperature above the normal optimum can damage a broad spectrum of cellular components leading to metabolic malfunction and ultimately cell death. Here, we further investigated the previously described observations that *C. metallidurans* CH34 exhibits a decreased survival frequency when cultivated at 37°C on rich medium (Mergeay et al., 1985; Van der Lelie et al., 1992; Sadouk and Mergeay, 1993; Mijnendonckx et al., 2013). This study revealed that all *C. metallidurans* strains tested displayed increased mortality on LB agar plates at 37°C, including more recently isolated strains such as those from human infection. The latter indicated that the TIMM phenomenon does not impede its survival and proliferation within the human body. Putatively, the environmental conditions, i.e. amino acids and ammonium concentrations, encountered do not induce TIMM (Hladky and Barrand, 2014; Simha and Ganesapillai, 2017). Furthermore, our observations reinforce the conservation of this phenotype in *C. metallidurans* strains, indicating the involvement of conserved cellular housekeeping processes and its use as a phylogenetic discriminatory trait. Cultivation of *C. metallidurans* CH34 on LB agar plates at 37°C indicated that the lower viable count at 37°C compared to 30°C resulted from induced mortality and not by reduced or stalled cell division.

The ability to induce TIMM was also tested for different inducers, and three different conditions were found that promoted mortality at the non-permissive temperature. It was shown that yeast extract and peptone, both containing a high peptide and amino acid content with yeast extract also comprising various vitamins and sugars, could separately promote TIMM. Moreover, it was demonstrated that both components promoted TIMM in at least two different ways as TIMM survivors resistant to peptone but not yeast extract were isolated. Alternatively, CA, another product containing small peptides and amino acids with the exception of tryptophan, promoted increased mortality at 37°C either as a sole carbon or nitrogen source or as an additional nutrient to MM284. In addition, seven different amino acids, namely, proline, cysteine, glycine, glutamine, leucine, histidine and phenylalanine, could induce TIMM. However, only phenylalanine promoted TIMM for all strains tested. Since different strain-specific inducers were found, the possible involvement of multiple and strain-specific pathways is proposed. In addition, increasing the level of inorganic nitrogen also induced mortality at 37°C. Second, Schatz medium, a mineral medium with low salt concentrations, also induced TIMM at 37°C. Schatz medium has a higher water activity than MM284 and thus renders the cell in a more hyperosmotic state where water will transport from the medium to the cell, putting pressure on the cell membrane. Third, *C. metallidurans* CH34 grown autotrophically also exhibited increased mortality at 37°C.

Scrutinizing protective agents able to inhibit TIMM showed that a complete mitigation of the TIMM phenotype was reached by the addition of a non-ionic osmolyte but not by ROS scavengers, indicating that ROS is not the primary or main cause of the observed mortality at 37°C. Addition of sorbitol to all known TIMM-promoting conditions protected the cells. A second protective factor for Schatz medium was increasing the osmolarity of the growth medium by the addition of salt approximating the level in MM284. However, the protective function of salt, but not of sorbitol, in Schatz medium was counteracted by the addition of CA. Thus, sorbitol exerts a general protective function to alleviate mortality of the cells at 37°C. Sorbitol has been shown to promote cell growth of *Zymomonas mobilis* in environments with high sugar concentration and had also a protective function under heat and ethanol stress, although the molecular mechanisms involved in tolerance to the stress conditions are still unclear (Loos et al., 1994; Sootsuwan et al., 2013). In yeast suffering cell wall defects, an osmotic stabilizer, i.e. sorbitol, was required for growth and proliferation, especially at elevated temperature, and it was postulated that cells might employ a non-lethal morphology checkpoint to monitor appropriate turgor pressure before undergoing further growth (Martin et al., 1993; Harrison et al., 2001; Hohmann, 2002). Increasing osmolarity of the growth medium or addition of sorbitol likely promotes cell membrane stability, which might be crucial for cell survival and further growth at 37°C.

Additional TIMM assays with *C. metallidurans* CH34 revealed a frequency of inherited stable TIMM resistance of 29%, with lower cross-resistance to other TIMM-inducing media, confirming the existence of multiple TIMM-promoting pathways. Two stable inherited *C. metallidurans* CH34 TIMM survivors showed multiple mutational changes, including insertions, deletions, point and frameshift mutations, and the redistribution of seven mobile genetic elements (MGEs), including IS*1088*, IS*Rme15*, IS*1090*, IS*1086*, IS*1087B* and Tn*6048*. Transposition frequency of IS elements can be promoted by stress challenges, and increased transposition of various IS elements under elevated temperature conditions was observed in *Burkholderia multivorans* ATCC 17616 (Ohtsubo et al., 2005). Furthermore, both TIMM survivors tested displayed loss of autotrophy, which was confirmed by a large 60-kb deletion comprising the *cbb*/*hox* locus, and this excision was most probably caused by IS*1071*-mediated deletion (Mijnendonckx et al., 2013). The many mutational changes observed in these two stable *C. metallidurans* CH34 TIMM survivors resemble the activation of a transient temperature-induced hypermutator phenotype somewhere during the selective condition.

One of the mutations shared by both TIMM survivors was a single-nucleotide substitution in Rmet_1009, resulting in a K62R substitution in the encoded oxidoreductase. As deletion of Rmet_1009 in the parental strain resulted in TIMM resistance, it is likely that this K62R substitution presents a loss-of-function mutation. The Rmet_1009 gene encodes for a RutE-like oxidoreductase, although not located in a *rutABCDEFG* operon as in *E. coli*. The *rut* operon, which is under the control of the nitrogen regulatory protein C (NtrC) (Zimmer et al., 2000), is involved in the degradation of exogenous pyrimidines as the sole nitrogen source (Loh et al., 2006; Kim et al., 2010). Interestingly, this degradation only occurs at room temperature and not at 37°C, probably due to the inadequate ability to remove toxic malonic semialdehyde at higher temperatures (Loh et al., 2006; Kim et al., 2010). Currently, we do not know if Rmet_1009, similar to RutE, reduces malonic semialdehyde to 3-hydroxypropionic acid and if *C. metallidurans* CH34 carries other genes that encode for proteins that enable the removal of malonic semialdehyde, similar to *Cupriavidus necator* H16 (Arenas-López et al., 2019). Nevertheless, our results point toward metabolic poisoning of *C. metallidurans* grown in high-concentration nitrogen-containing media at 37°C.

## Data Availability Statement

The datasets presented in this study can be found in online repositories. The names of the repository/repositories and accession number(s) can be found below: https://www.ncbi.nlm.nih.gov/, PRJNA641768.

## Author Contributions

RV and AA: conceptualization, writing–review and editing, and supervision. RV, JV, and AA: methodology. RV, JV, WH, PM, and AP: validation, formal analysis, and investigation. RV: data curation and visualization. RV and JV: writing original draft preparation. NL: project administration. All authors have read and agreed to the published version of the manuscript.

## Conflict of Interest

The authors declare that the research was conducted in the absence of any commercial or financial relationships that could be construed as a potential conflict of interest.

## Supplementary Material

Supplementary Table 1 Primers used in this study.
