# Supplementary material for: Phenotypic and Genetic Characterization of Temperature-Induced Mutagenesis and Mortality in Cupriavidus metallidurans
Source: Front Microbiol. 2021 Jul 9;12:698330. doi: 10.3389/fmicb.2021.698330 (PMC8299993; doi:10.3389/fmicb.2021.698330)
Supplement: Supplementary Table 1 — Primers used in this study. [file Table_1.DOCX]

Supplementary Material

# Supplementary Figures and Tables

Supplementary Table 1. Primers used in this study.

| pK18mob_FW | CTGGCGTAATAGCGAAGAGG |
| --- | --- |
| pK18mob_RV | TATCCGCTCACAATTCCACA |
| Rmet_1009_3’_FW | GATCACTAGTCCTGACGTTCGACGAAGCC |
| Rmet_1009_3’_RV | CTAGGAATTCGAGCGCATGGAAGCGAAAGC |
| Rmet_3139_5’_FW | ACAGATGCATCGCCAGGTGCAG |
| Rmet_3139_5’_RV | TTATCTTAAGTGTGCAAGGACTGCCTGATC |
| Tet _FW | GATCACTAGTTCAGCCCCATACGATATAAG |
| Tet_RV | TTATCTTAAGTGGAGTGGTGAATCCGTTAG |

^1^ Restriction sites are underlined.
